# Clinical application research on the quantitative measurement of supraspinatus muscle fatty degeneration based on PACS system to improve preoperative assessment

**DOI:** 10.3389/fsurg.2025.1627901

**Published:** 2025-07-02

**Authors:** Sitong Zhang, Yan Huang, Jian Hu, Shiao Li, Beijie Qi, Wu Wang, Menghong Cao, Qian Wang

**Affiliations:** ^1^ Department of Orthopedics, Shanghai Pudong Hospital, Fudan University Pudong Medical Center, Shanghai, China; ^2^ Department of Rehabilitation, Shanghai Pudong Hospital, Fudan University Pudong Medical Center, Shanghai, China; ^3^ Department of Medical Imaging, Shanghai Pudong Hospital (Fudan University Pudong Medical Center), Shanghai, China

**Keywords:** fatty infiltration, PACS (picture archiving and communication system), preoperative assessment, rotator cuff tear (RCT), supraspinatus (SSP)

## Abstract

**Objective:**

To evaluate the clinical efficacy of a novel quantitative method using the Picture Archiving and Communication System (PACS) for multiplane assessment of supraspinatus muscle fatty infiltration (FI) and compare its reliability and accuracy with traditional single-plane visual evaluations (Under Direct Vision-FF) in preoperative planning for rotator cuff tear (RCT) patients.

**Methods:**

A retrospective analysis was conducted on patients undergoing arthroscopic rotator cuff repair (ARCR) between January and June 2023. Preoperative 3.0 T MRI scans were analyzed using PACS to measure FI in three sagittal planes (medial, Y-plane, lateral). Four orthopedic surgeons performed Goutallier classification and manual FI assessments under direct vision and via PACS. Intra- and interobserver reliability were evaluated using intraclass correlation coefficients (ICCs), while Bland-Altman analysis and paired *t*-tests compared measurement consistency and differences.

**Results:**

PACS-based measurements (PACS-FF) demonstrated superior reliability (intraobserver ICC: 0.973–0.996; interobserver ICC: 0.940–0.978) compared to direct vision assessments (intraobserver ICC: 0.538–0.967; interobserver ICC: 0.864–0.940). Significant discrepancies were observed between methods, with direct vision underestimating FI (*p* < 0.05–0.0001). Multiplane analysis revealed heterogeneous FI distribution, with lateral-plane FI significantly higher than medial and Y-plane values (*p* < 0.001). Bland-Altman analysis showed 60%–85% of direct vision measurements exceeded clinically acceptable limits of agreement (±10%).

**Conclusions:**

Quantitative multiplane PACS-based FI assessment improves accuracy and reliability over traditional single-plane visual evaluation, better reflecting heterogeneous fat distribution in the supraspinatus muscle. This method enhances preoperative risk stratification and surgical outcome prediction for RCT patients. Future integration of automated tools may further optimize clinical efficiency.

## 1 Introduction

Rotator cuff injury is one of the most prevalent disorders of the musculoskeletal system, primarily characterized by shoulder pain and functional impairment. Epidemiological studies have demonstrated that the incidence of rotator cuff injury increases progressively with age (1, 2). For patients who do not respond to conservative treatment, surgical intervention often becomes the preferred therapeutic strategy. Arthroscopic rotator cuff repair (ARCR) is currently the most commonly employed surgical technique for treating rotator cuff tears in clinical practice. By reattaching the torn tendon to the proximal humerus using sutures, ARCR can yield satisfactory clinical outcomes (3–7).

However, postoperative re-tear remains a significant concern, particularly in cases involving large to massive rotator cuff tears, where the re-tear rate may be as high as 94% (6, 8). Tendon repair failure can result in unfavorable clinical outcomes and may even increase the risk of secondary osteoarthritis (9). Multiple studies have established a strong association between the degree of preoperative rotator cuff fatty infiltration and the risk of postoperative re-tear. Specifically, rotator cuffs with more advanced fatty infiltration are significantly more likely to experience re-tear than those with minimal infiltration (2, 10). Recent research has shown that fatty infiltration of the supraspinatus muscle is an independent risk factor for rotator cuff re-tear. The severity of muscle fatty infiltration is closely related to the risk of re-tear, and this correlation is particularly significant in the supraspinatus (11, 12).

Fatty infiltration and muscle atrophy are both common pathological changes in rotator cuff injuries. Therefore, accurate preoperative evaluation of fatty infiltration is critical for optimizing treatment planning, predicting surgical outcomes, and guiding postoperative rehabilitation. The grading system for fatty infiltration was nitially proposed by Goutallier et al. in 1994 based on axial computed tomography (CT) imaging. This system classifies fatty infiltration into five stages: Stage 0 indicates normal muscle without fatty infiltration; Stage 1 shows a few intramuscular fat streaks; Stage 2 indicates more muscle than fat; Stage 3 represents an equal amount of muscle and fat; and Stage 4 is characterized by more fat than muscle. With advances in magnetic resonance imaging (MRI), Fuchs et al. adapted this classification system for use with MRI, which has since become widely accepted in clinical practice (13, 14).

Currently, fatty infiltration is most commonly assessed using the Goutallier classification (GC), which evaluates fatty infiltration semi-quantitatively on scapular Y-view images from oblique sagittal MRI scans. The scapular Y-view refers to the characteristic Y-shaped appearance of the scapula and spine on oblique sagittal MRI slices (15). However, this method is primarily based on single-slice, semi-quantitative evaluation, which relies heavily on the anatomical consistency of the selected image. This single-slice approach fails to account for regional variations in muscle morphology and may therefore limit the accuracy of fatty infiltration assessment. Additionally, due to its semi-quantitative nature, the GC system is highly susceptible to interobserver variability, with different evaluators potentially arriving at inconsistent results. Previous studies have reported poor interobserver reliability for this method (16, 17).

As a result, there is a need for more accurate and objective techniques to evaluate fatty infiltration. In previous work, Vidt et al. compared GC-based fatty infiltration measurements in scapular Y-view slices with three-dimensional (3D) quantitative assessments of the entire rotator cuff muscle. They found that single-slice evaluations did not adequately reflect the 3D distribution of fatty infiltration (18). Nevertheless, while 3D methods offer improved accuracy, they are often limited in clinical settings due to their technical complexity and operational demands.

To improve upon existing limitations, recent research has explored advanced MRI techniques for quantifying muscle fat content. Notably, chemical shift–based water-fat separation imaging techniques, such as IDEAL and Dixon, have shown considerable promise. These quantitative MRI methods allow for objective, continuous measurement of fat content, offering superior volumetric accuracy compared to traditional approaches. However, their widespread clinical use is hindered by the need for specialized imaging sequences, increased scanning time, and higher costs—factors that restrict implementation in routine clinical practice, especially in high-throughput or resource-limited settings (19–22).

Based on this background, the present study proposes a novel method for the quantitative assessment of rotator cuff fatty infiltration. This method involves measuring muscle fat content directly using the Picture Archiving and Communication System (PACS) to obtain a more precise evaluation. Specifically, three sagittal MRI slices encompassing the scapular Y-view—comprising the central Y-view image and its adjacent medial and lateral slices—were selected. Fat content was measured in each of the three slices, and the average value was calculated to serve as a quantitative indicator of muscle fatty infiltration. The study further compared this multiplanar average with conventional single-slice measurements to assess the feasibility and accuracy of this novel approach.

## 2 Methods

### 2.1 Patient selection and evaluation

**Figure 1:**
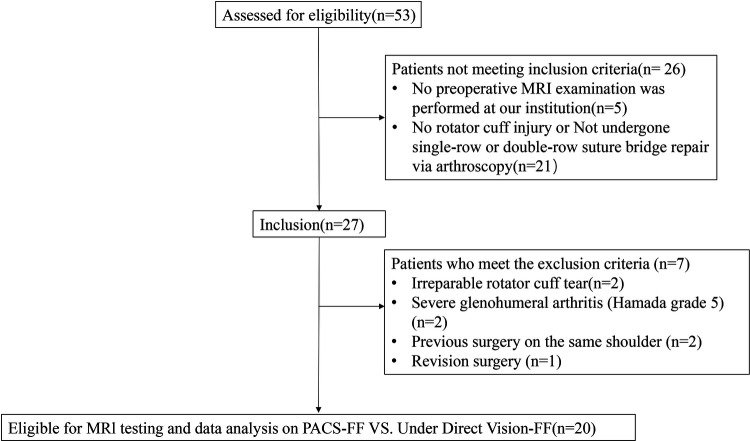
Inclusion and exclusion criteria.

This study is a retrospective analysis involving patients who underwent arthroscopic rotator cuff repair (ARCR) at the author's institution between January 2023 and June 2023. The study protocol was approved by the institutional review board. All patients signed a standardized informed consent form during their hospital stay, stating that their medical data might be used for educational and research purposes. Patients who had undergone arthroscopic shoulder surgery were included in the study if they met the following criteria: (1) preoperative magnetic resonance imaging (MRI) was performed at our institution; (2) they underwent arthroscopic single-row or double-row suture bridge repair; (3) partial or full-thickness tears of the supraspinatus tendon that were repairable. Exclusion criteria included: (1) irreparable rotator cuff tear; (2) severe glenohumeral arthritis (Hamada grade 5); (3) previous surgery on the same shoulder; (4) revision surgery; (5) loss to follow-up (Figure 1).

### 2.2 MRI protocol and evaluation

MRI examination was performed in all patients using a 3.0 T magnet (GE DISCOVERY MR750W) with an interval of 4 mm for each sagittal slice (3.0 T scanner; echo time = 68 ms; repetition time = 1,822 ms; flip angle = 111°; matrix size = 288 × 256; bandwidth = 289 kHz; field of view = 150 mm; slice thickness = 4 mm). The total scan time was approximately 15 min (3 T). Measurements were conducted using the Picture Archiving and Communication System (PACS) program (Kingstar Winning TWebView 2012) provided by the medical center.

### 2.3 Assessment of the goutallier classification by MRI

**Figure 2:**
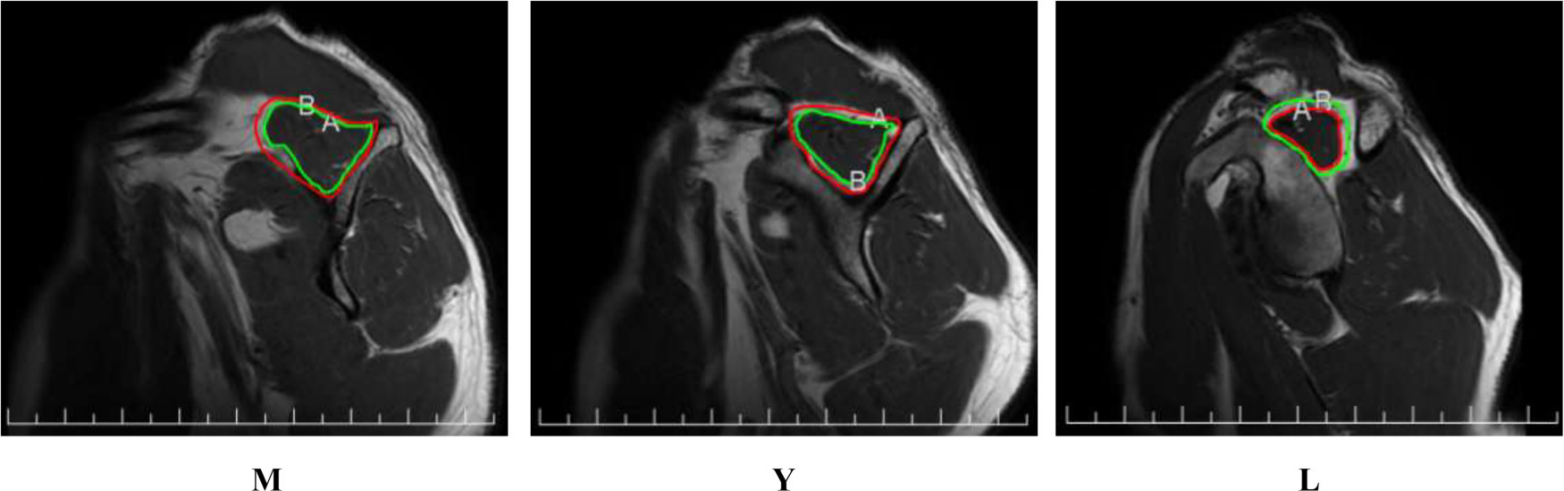
Three-slice levels in a shoulder magnetic resonance imaging oblique sagittal view. (M) The medial mid-slice (Y) The scapular Y-view (L) The lateral mid-slice [In the figures, the areas A (the area within the green line) and B (the area within the red line) were delineated using the PACS system. “A” denotes the residual muscle area, while “B” represents the total area of the supraspinatus muscle.].

Firstly, four orthopedic surgeons (a junior resident, a senior resident, an attending physician, and a chief physician) independently assessed the Goutallier grade (GY) of the scapular Y-view images on oblique sagittal MRI scans of the shoulders of in each subject with rotator cuff injury. Subsequently, the four surgeons conducted two separate assessments for the same group of subjects, with a 2-week interval between the evaluations. A three-plane Goutallier classification was then performed. We assessed the Goutallier grade on three sagittal planes. Based on the scapular Y-view, we defined three measurement planes, which included the scapular Y-view and the approximate mid-slices on the medial and lateral sides of the Y-view. Due to individual variations in shoulder anatomy and size, the number of slices obtained medially and laterally from the Y-view varied among subjects. To standardize the measurements, in the Y-view imaging of the shoulder MRI, the medial mid-slice was defined as the third slice located 8 mm medial to the Y-view, which is situated in the medial part of the scapula and is primarily used to evaluate the muscles and fatty infiltration on the medial side of the shoulder. The lateral mid-slice was defined as the fourth or fifth slice located 12 mm or 16 mm lateral to the Y-view, which displays the midpoint of the Y-view and the lateral end of the supraspinatus tendon, and is primarily used to evaluate the muscles and fatty infiltration on the lateral side of the shoulder (Figure 2). The Goutallier grade of the medial mid-slice was recorded as GM, and that of the lateral mid-slice was recorded as GL. The average Goutallier grade (GA) was calculated by taking the mean of the Goutallier grades from the three planes.

### 2.4 Fi ratios measured from MRI examination

In this study, a standardized procedure was employed to assess the fatty infiltration (FI) score of the supraspinatus muscle. Initially, the four aforementioned orthopedic surgeons visually evaluated the degree of fatty infiltration of the supraspinatus muscle on the scapular Y-view, as well as on the medial and lateral slices of the oblique sagittal shoulder MRI, and recorded their preliminary estimates, denoted as FIY, FIM, and FIL, respectively. Subsequently, each surgeon manually delineated the contour of the supraspinatus muscle on the three oblique sagittal slices using the PACS system. The region of interest (ROI) for the supraspinatus muscle was defined as a 1–2 mm range from the muscle boundary and fascia, as determined by visual inspection of the T2-weighted imaging, to ensure that the peripheral fat of the muscle was excluded from the ROI (Figure 2). By outlining the entire extent of the supraspinatus muscle and its muscular component, the ROI of the supraspinatus muscle (Ss) and the residual muscle area (Sm) were obtained, respectively. The difference between Ss and Sm was calculated as the area of fatty infiltration in the supraspinatus muscle, and the ratio of Sf to Ss (Sf/Ss) was defined as the fatty infiltration score (fi) of the supraspinatus muscle. The value of fi multiplied by 100 was defined as the Fat Fraction (%). [Sf = Ss − Sm;fi = Sf/Ss; Fat Fraction (%) = fi × 100]. The fi value measured on the scapular Y-view was denoted as fiY. Similarly, the aforementioned procedure was repeated on the medial mid-slice and lateral mid-slice to obtain fiM and fiL, respectively. Finally, the average value of fiY, fiM, and fiL was calculated to obtain the final fiA value. Each evaluator performed two independent measurements with a 2-week interval to ensure the reliability and reproducibility of the measurement results. All images were anonymized and randomized to maintain the objectivity and impartiality of the assessments.

### 2.5 Statistical analysis

Descriptive quantitative data are presented as mean ± standard deviation (SD), while qualitative data are presented as counts and percentages. Statistical analyses were performed using SPSS software or Prism 10 (GraphPad Software). Statistical significance was set at *P* < 0.05. The statistical significance of the mean differences between the fatty infiltration scores obtained under direct vision (Under Direct Vision-FF) and those measured using the PACS system (PACS-FF) was assessed using paired t -tests. Bland-Altman plots were used to display the percentage differences (D%): (Measurement A − Measurement B)/mean of the two measurements × 100. The limits of agreement (LOA) were calculated as Mean ± 1.96SD; clinically acceptable LOA were defined as ±10% (23). Intraobserver and interobserver reliability were evaluated using intraclass correlation coefficients (ICCs). The statistical significance of mean differences in fatty infiltration scores between different planes was also assessed using paired t -tests. ICC values were interpreted as follows: ICCs < 0.50 = poor, 0.50–0.75 = moderate, > 0.75–0.90 = good, and >0.90 = excellent (23, 24).

## 3 Results

Between January 2023 and June 2023, 53 patients underwent arthroscopic shoulder surgery at our institution. Of these, 33 patients were excluded for the following reasons: absence of preoperative MRI performed at our institution (*n* = 5), no supraspinatus tear or not treated with single-row or double-row suture bridge technique (*n* = 21), irreparable rotator cuff tear (*n* = 2), severe glenohumeral arthritis (Hamada grade 5) (*n* = 2), previous surgery on the same shoulder (*n* = 2), and revision surgery (*n* = 1). The final study cohort consisted of 20 patients who met the inclusion criteria for arthroscopic rotator cuff repair (ARCR), including 7 males and 13 females, with a mean age of 60.40 ± 13.63 years.

### 3.1 Differences between under direct vision-Ff and PACS-Ff measurements

**Figure 3:**
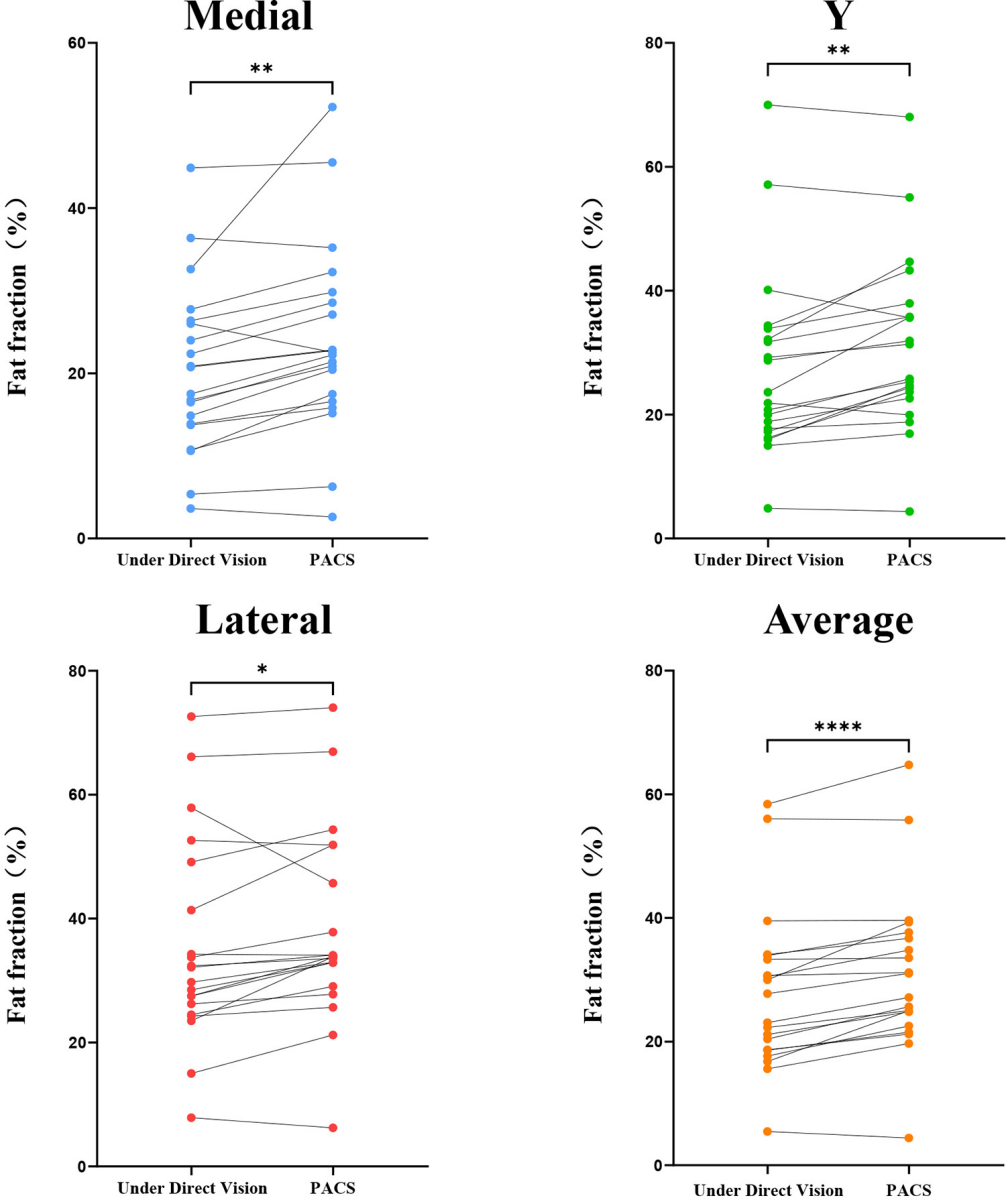
Paired *t*-tests for under direct vision-FF and PACS-FF in the medial, Y-plane, and lateral supraspinatus muscle.

**Figure 4:**
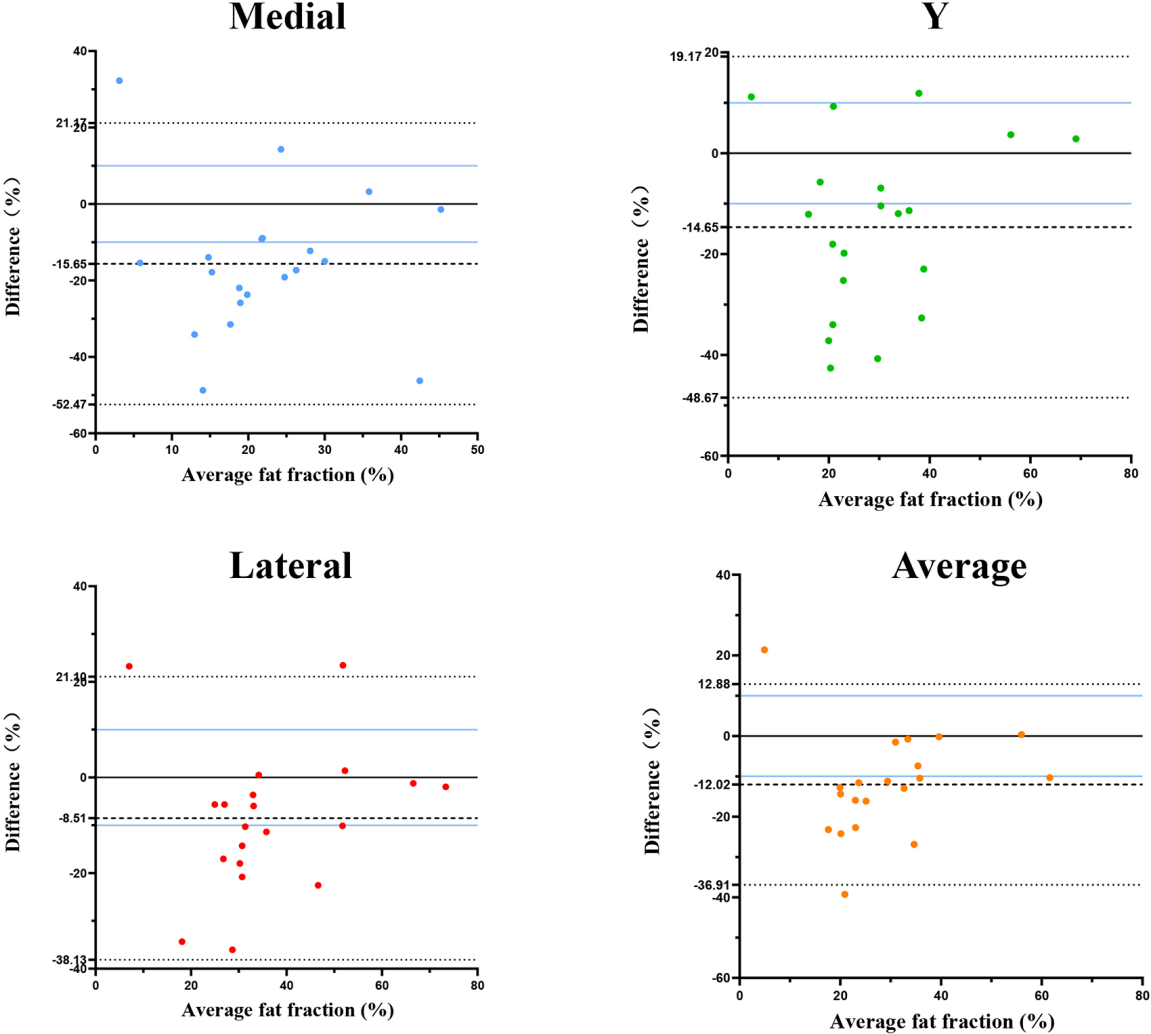
Bland-Altman plot of agreement between under direct vision-FF and PACS-FF of the supraspinatus in medial (M), Y-plane (Y), and lateral (L) planes in rotator cuff tears. Under Direct Vision-FF, Under Direct Vision fat fraction; PACS-FF, PACS fat fraction; LOA, limit of agreement.

When measuring the fat fraction in three different planes (M, Y, L) and their averages, the Under Direct Vision-FF values were higher than the PACS-FF in most patients. The results of the Paired *t*-tests demonstrated that Under Direct Vision-FF was significantly higher than PACS-FF in all three measurement planes and their averages: M plane (*p* < 0.002), Y plane (*p* < 0.002), L plane (*p* < 0.05), and the average (*p* < 0.0001). In terms of the average measurements, Under Direct Vision-FF was lower than PACS-FF in 90% of average measurements; for the medial measurements, Under Direct Vision-FF was lower than PACS-FF in 85% of cases; for the Y-plane measurements, Under Direct Vision-FF was lower than PACS-FF in 75% of cases; and for the lateral measurements, Under Direct Vision-FF was lower than PACS-FF in 85% of cases (Figure 3). Furthermore, the Bland-Altman plot showed poor agreement between Under Direct Vision-FF and PACS-FF. In most cases, the differences between Under Direct Vision-FF and PACS-FF exceeded the clinically acceptable limits of agreement (LOA). For the average, medial, Y-plane, and lateral measurements, 16 (80%), 17 (85%), 15 (75%), and 12 (60%) data points, respectively, fell outside the LOA (Figure 4).

### 3.2 Differences in FF values among various measurement planes

**Figure 5:**
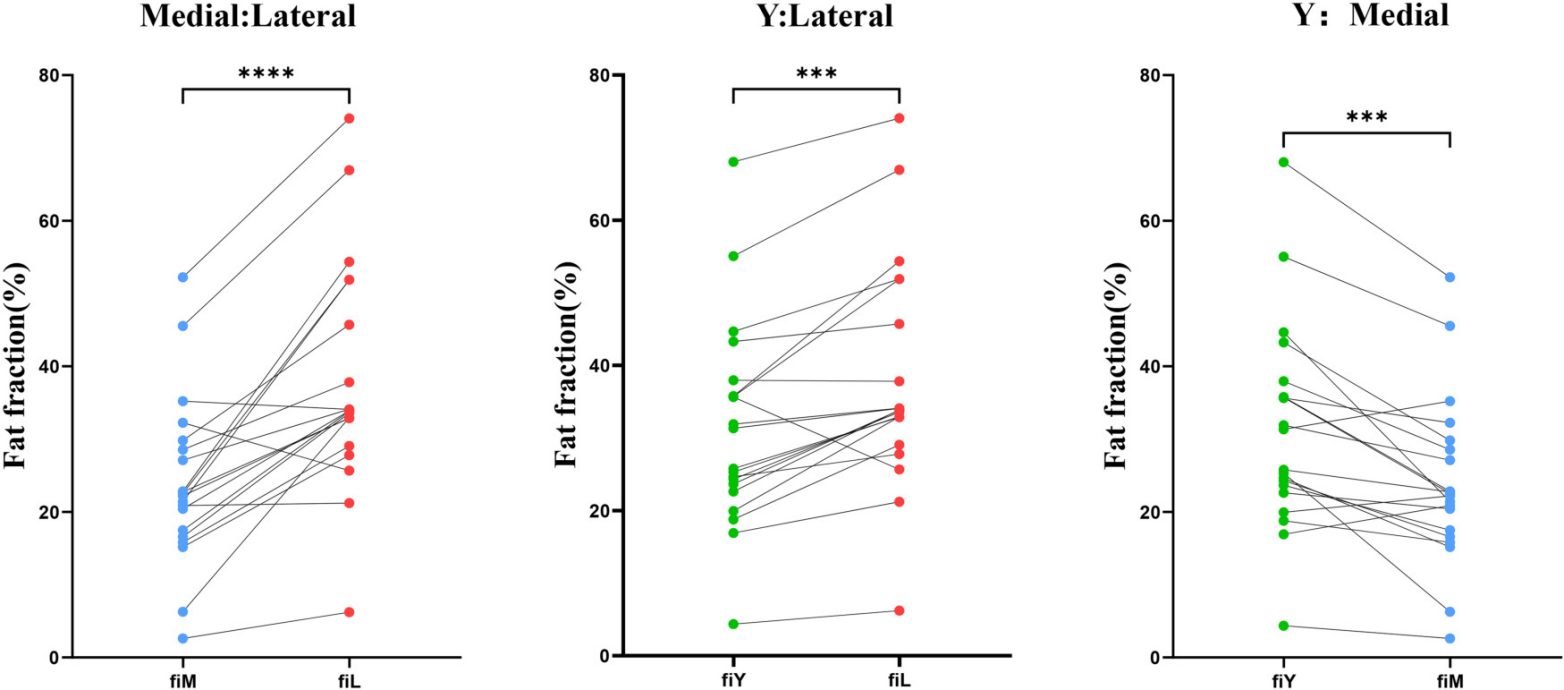
Paired *t*-tests were used to compare the fat fractions in the medial, Y-plane, and lateral aspects of the supraspinatus muscle.

In all patients, the fat infiltration fraction (FF) of the supraspinatus muscle was measured in three different planes (lateral, Y-plane, and medial), and the results showed a substantial heterogeneity in the distribution of fat within the supraspinatus muscle. This finding indicates that the fat infiltration fraction measured in a single plane may not accurately reflect the degree of fat infiltration in the entire supraspinatus muscle. Further paired *t*-test analysis revealed that the degree of fat infiltration in the supraspinatus muscle at the lateral plane was significantly higher than that at the medial plane (*p* < 0.001) and the Y-plane (*p* < 0.001), and fat infiltration at the Y-plane was also significantly higher than that at the medial plane (*p* < 0.001) (Figure 5). These results indicate significant interplanar differences in the degree of fat infiltration in the supraspinatus muscle across different planes, underscore the importance of multiplane measurements in comprehensively assessing fat infiltration in the supraspinatus muscle.

### 3.3 Intra- and inter-observer reliability of under direct vision-FF and PACS-FF measurements

In this study, the intra- and inter-observer reliability of the fat fraction (FF) measurements obtained under direct vision (Under Direct Vision-FF) and via the PACS system (PACS-FF) was evaluated. The results showed that the intra-observer reliability of Under Direct Vision-FF (ICC range: 0.538–0.967) was moderate to excellent, but lower than that of PACS-FF (ICC range: 0.973–0.996) (Tables 1, 2). Additionally, the inter-observer reliability of Under Direct Vision-FF (ICC range: 0.864–0.940) was significantly lower than that of PACS-FF (ICC range: 0.940–0.978) (see Table). These results indicate that PACS-FF exhibits greater reliability and consistency than Under Direct Vision-FF in assessing fatty infiltration of the supraspinatus muscle.

**Table 1:** Intraobserver agreement of uder direct vision-FF and PACS-FF.

Plane	Observer1				Observer2				Observer3				Observer4			
	Under direct vision		PACS		Under direct vision		PACS		Under direct vision		PACS		Under direct vision		PACS	
	ICC	95%CI	ICC	95%CI	ICC	95%CI	ICC	95%CI	ICC	95%CI	ICC	95%CI	ICC	95%CI	ICC	95%CI
Medial	0.719	0.415–0.878	0.996	0.990–0.998	0.641	0.289–0.841	0.985	0.963–0.994	0.747	0.463–0.891	0.982	0.955–0.993	0.538	0.137–0.787	0.992	0.980–0.997
Y	0.967	0.918–0.987	0.980	0.949–0.992	0.718	0.413–0.878	0.995	0.988–0.998	0.846	0.651–0.936	0.990	0.976–0.996	0.795	0.555–0.914	0.992	0.981–0.997
Lateral	0.845	0.650–0.936	0.973	0.933–0.989	0.743	0.456–0.890	0.979	0.947–0.991	0.858	0.677–0.941	0.990	0.975–0.996	0.755	0.479–0.895	0.987	0.967–0.995
Average	0.930	0.831–0.972	0.993	0.982–0.997	0.895	0.754–0.957	0.993	0.983–0.997	0.844	0.648–0.935	0.994	0.985–0.998	0.867	0.696–0.945	0.995	0.987–0.998

**Table 2:** Interobserver agreement of uder direct vision-FF and PACS-FF.

Plane	Under direct vision		PACS	
	ICC	95%CI	ICC	95%CI
Medial	0.864	0.755–0.936	0.940	0.886–0.973
Y	0.932	0.871–0.969	0.980	0.960–0.991
Lateral	0.904	0.822–0.956	0.963	0.929–0.983
Average	0.940	0.887–0.973	0.978	0.957–0.990

### 3.4 Intra- and inter-observer reliability of the Goutallier classification

Among the four observers, the intra-observer reliability of the Goutallier classification (ICC range: 0.908–1.000) was excellent, while the inter-observer reliability (ICC range: 0.876–0.947) was good to excellent. These results indicate that the Goutallier classification demonstrates high reliability in assessing fatty infiltration of the supraspinatus muscle (Tables 3, 4).

**Table 3:** Intraobserver agreement of goutallier.

Plane	Observer1		Observer2		Observer3		Observer4	
	ICC	95%CI	ICC	95%CI	ICC	95%CI	ICC	95%CI
Medial	0.953	0.866–0.981	1	1	0.884	0.730–0.952	1	1
Y	1	1	0.946	0.870–0.978	0.855	0.670–0.940	0.948	0.874–0.979
Lateral	0.966	0.916–0.986	0.908	0.782–0.962	0.973	0.934–0.989	0.975	0.938–0.990
Average	0.974	0.935–0.989	0.974	0.935–0.989	0.929	0.829–0.971	0.988	0.970–0.995

**Table 4:** Interobserver agreement of goutallier.

Plane	ICC	95%CI
Medial	0.876	0.775–0.943
Y	0.947	0.900–0.976
Lateral	0.919	0.849–0.963
Average	0.945	0.896–0.975

## 4 Discussion

The relationship between rotator cuff muscle fatty infiltration and postoperative re-tear rates, as well as shoulder function, has been well established by numerous studies (25–27). However, the accuracy of current methods used to assess rotator cuff muscle fatty infiltration remains a key topic of discussion among researchers. Most previous studies have relied on Goutallier grading or fat fraction measurement using a single imaging plane, which often fails to provide a comprehensive assessment of the entire supraspinatus muscle. As a result, such evaluations may not accurately reflect the overall muscle quality. Moreover, assessments based on a single slice are highly dependent on the evaluator's experience and the selected section. In routine clinical practice, judgments regarding the severity of supraspinatus muscle fatty infiltration are often made under direct visual estimation—a method that is subjective and lacks both objectivity and accuracy (13, 14, 28–31). Therefore, this study compared measurements obtained using the PACS system with those made under direct visual inspection, aiming to highlight the advantages of PACS-based evaluation in clinical settings. In addition to the traditional scapular Y-view, medial and lateral mid-slices were incorporated to achieve a more comprehensive multiplanar assessment. The results revealed a heterogeneous distribution of fat across the supraspinatus muscle. This supports the view that evaluation based on a single plane is insufficient for capturing the true extent of fatty infiltration.

To our knowledge, few studies have conducted quantitative multiplanar evaluations of supraspinatus fatty infiltration using the PACS system. This study employed a robust analytical approach, including numerical comparisons, Bland–Altman agreement analysis, and intra- and inter-observer reliability assessments. The most important finding was the significant discrepancy between the two assessment methods: measurements obtained under direct vision consistently yielded lower fat fraction values than those obtained using PACS. Notably, in 60% to 85% of cases, the difference exceeded the clinically acceptable margin of 10%, suggesting that direct visual assessment may lead to underestimation of the true fat content.

A review of the measurement process revealed that fatty infiltration of the muscle often progresses from the periphery of the muscle to the interior. Therefore, in the evaluated images, for example, in the oblique sagittal section, fat tissue is predominantly located in the outer layers of the muscle, while muscle tissue is distributed as a whole in the center of the supraspinatus fossa. Consequently, when measuring under direct vision, the area of peripheral fat tissue is often underestimated, while the area of internal muscle tissue is overestimated, resulting in an underestimation of the degree of fatty infiltration under direct vision.

Previous studies have demonstrated that preoperative fatty infiltration is a critical prognostic factor affecting both surgical outcomes and functional recovery (13, 14, 32). Therefore, accurate preoperative evaluation is essential for effective surgical planning and outcome prediction.

It is worth noting that differences in the degree of fatty infiltration across different planes may be related to the uneven distribution of fat within the muscle following rotator cuff tears (29). In some basic scientific research studies, the rationale for using a single image to assess fatty infiltration throughout the entire muscle has been challenged. In clinical and animal studies, infiltrated fat has been shown to be predominantly distributed near the site of the tear rather than throughout the entire muscle belly (33–36). Similarly, in the measurements taken in this study, the degree of fatty infiltration in the lateral muscle was significantly greater than that in the traditional Y-plane and medial plane. Particularly in cases of muscle injury, the retraction of the tendon-muscle junction may lead to the conclusion that assessing the degree of fatty infiltration in the supraspinatus muscle based on a single plane is insufficient to represent the severity of fatty infiltration throughout the entire supraspinatus muscle.

Another important finding is the excellent reliability of PACS measurements, especially in terms of inter-observer consistency. The intra- and inter-observer reliability of PACS system measurements (intra-class correlation coefficients, ICCs, ranging from 0.973 to 0.996 and 0.940 to 0.978, respectively) was significantly superior to that of direct vision (ICCs ranging from 0.538 to 0.967 and 0.864 to 0.940, respectively). This result indicates that the assessment of fatty infiltration using the PACS system is more reproducible and reliable than that made under direct vision.

Interestingly, regardless of whether the assessment of fatty infiltration was made under direct vision or using the PACS system, the intra- and inter-observer consistency in the Y-plane was superior to that in the medial and lateral planes. The reason may be that the Y-plane is the section that displays the largest area of the supraspinatus fossa. The supraspinatus muscle is relatively regular in this plane, especially in cases with severe fatty infiltration, where the shape of the supraspinatus muscle in the Y-plane is more regular compared to the other two planes. This finding also corroborates, from another perspective, why the degree of fatty infiltration in the supraspinatus muscle is traditionally assessed in a single Y-plane.

Moreover, by comparing the intra-class correlation coefficients for intra- and inter-observer measurements using the PACS system, we found that the inter-observer ICC values were somewhat lower. This phenomenon may be related to differences among measurers in delineating the extent of the supraspinatus muscle during the measurement process. During the measurement process, we found that there was little difference among measurers in the measurement values of the internal muscle area, but there were significant differences in the measurement values of the entire supraspinatus muscle extent. In normal supraspinatus muscles, the boundary between the supraspinatus muscle and surrounding bone and soft tissues is relatively clear, which allows for more accurate definition of the region of interest (ROI) and measurement of fatty infiltration. However, in supraspinatus muscles with fatty infiltration or tears, the boundary between the muscle and surrounding tissues is unclear due to the interference of surrounding inflammation, edema signals, and muscle retraction itself, which affects the delineation of the ROI and, in turn, affects the assessment of the Goutallier classification and fatty infiltration of the supraspinatus muscle. Therefore, how to accurately define the exact extent of the supraspinatus muscle remains an important problem that needs to be solved.

In addition, we also conducted an observer consistency analysis of the Goutallier classification. The results showed that the intra- and inter-observer consistency (intra-class correlation coefficients, ICCs, ranging from 0.908 to 1.000 and 0.876 to 0.947, respectively) indicated that the Goutallier classification has good reproducibility and reliability across different planes. However, similar to the differences in the degree of fatty infiltration of the supraspinatus muscle measured in different planes using the PACS system, the Goutallier classification also showed significant differences across different planes. This result once again highlights the uneven distribution of fat in torn muscles, and suggests that a single plane cannot represent the degree of fatty infiltration throughout the entire supraspinatus muscle.

Existing studies have shown that preoperative fatty infiltration is an important prognostic factor affecting surgical outcomes and functional recovery (13, 37–39) In a retrospective study by Melis et al., a total of 1,668 patients were included, and the results showed that the higher the degree of fatty infiltration of the supraspinatus muscle, the earlier the symptoms appeared. The study pointed out that rotator cuff repair surgery should be performed before fatty infiltration occurs, to avoid muscle quality degradation and poor repair outcomes due to fatty infiltration (40). In addition, a study by Goutallier et al. involving 220 patients with open rotator cuff tears repaired using the bone tunnel suture technique found that when the Global Fatty Degeneration Index (GFDI) exceeded 2, the re-tear rate significantly increased (36). A review article by David et al. also pointed out that an increase in the degree of fatty infiltration is closely related to a higher re-tear rate and worse functional prognosis (41). These studies all emphasize the important role of fatty infiltration in anatomical failure and functional recovery after rotator cuff repair surgery.

The Goutallier score was originally used to estimate the proportion of fatty infiltration using a single CT slice (13, 14). Traditionally, the scapular Y-plane slice in oblique sagittal MRI of the shoulder has been widely used to assess the degree of fatty infiltration in the supraspinatus muscle. However, in recent years, studies have questioned the effectiveness of this method (40, 42). Studies have shown that the traditional single-slice method may not accurately reflect the degree of fatty infiltration throughout the entire supraspinatus muscle and may even produce misleading results. Similar to our study findings, although the traditional Y-plane has reliable bony landmarks, it fails to capture morphological changes in other locations, especially at the tendon-muscle junction. Therefore, the relatively lateral position of the scapular Y-plane slice may not well represent the entire muscle.

In addition, the lateral slice may overestimate fatty infiltration in the rotator cuff muscle, and when the rotator cuff muscle is injured, the slice may be affected by the retraction of the tendon-muscle junction. Studies have also shown that the distribution of fatty infiltration in the muscle belly is non-uniform, which also questions the rationale for using a single image to assess fatty infiltration (12, 42–46). Vidt et al. questioned whether the traditional single-slice method can accurately reflect the degree of fatty infiltration throughout the entire supraspinatus muscle. They believe that the traditional method may lead to biased measurement results. The main reason is that the relatively lateral position of the scapular “Y” plane slice may not fully represent the degree of fatty infiltration in the entire supraspinatus muscle. In addition, slices close to the scapula may overestimate fatty infiltration in the rotator cuff muscle during measurement. Moreover, when the rotator cuff muscle is injured, the slice may also be affected by the retraction of the tendon-muscle junction, which further affects the accuracy of the measurement results (18).

It can be seen that single-plane fatty infiltration measurements may lead clinicians to erroneous conclusions. Compared with single-plane assessment of fatty infiltration in the supraspinatus muscle, multiplane assessment is particularly important in clinical settings. Multiplane assessment can reflect the degree of infiltration in different parts of the supraspinatus muscle and better represent the degree of infiltration throughout the entire muscle. Therefore, multiplane measurement of fatty infiltration using the PACS system can better reflect the overall muscle quality. At the same time, because the PACS system has high intra- and inter-observer reliability, its measurements significantly reduce subjective bias. Thus, measuring fatty infiltration across multiple planes of the supraspinatus muscle using the PACS system is of greater clinical significance.

However, in a busy clinical setting, quantitative measurement of multiplanar fatty infiltration using the PACS system not only increases the workload of clinicians but also requires diagnosticians to have a clear understanding of shoulder anatomy on MRI. Moreover, PACS-based measurements currently only assess the degree of fat infiltration in the outer areas of the muscle and are unable to accurately measure fat infiltration within the muscle itself.

## 5 Conclusion

Quantitative multiplane PACS-based FI assessment significantly improves accuracy and reliability in detecting heterogeneous fat distribution within the supraspinatus muscle compared to traditional single-plane visual evaluation. This enhanced diagnostic capability translates to better preoperative risk stratification and surgical outcome prediction for rotator cuff tear (RCT) patients. ​However, the adoption of this more complex method must be balanced against the practical challenges it presents in clinical workflows, including potentially increased resource requirements, time commitment, and the need for specialized expertise or technology. Future developments, such as the integration of automated tools, hold promise for mitigating these challenges and optimizing the method's clinical efficiency.

## Data Availability

The raw data supporting the conclusions of this article will be made available by the authors, without undue reservation.
